# Addition of daratumumab to standard triplet regimens achieved better survival in newly diagnosed multiple myeloma: a systematic review and meta-analysis of randomized controlled trials

**DOI:** 10.3389/fonc.2025.1619115

**Published:** 2025-10-30

**Authors:** Bin Hu, Jun Wang, Dan Fang, Ling Jiang, Tianqi Li, Jinxia Cao

**Affiliations:** Department of Hematology, Changde Hospital, Xiangya School of Medicine, Central South University, The First People’s Hospital of Changde City, Changde, China

**Keywords:** daratumumab, quadruplet regimens, triplet regimens, multiple myeloma, meta-analysis

## Abstract

**Background:**

Triplet regimens, such as bortezomib-lenalidomide-dexamethasone (VRd) and bortezomib-melphalan-prednisone (VMP), were standard treatments for newly diagnosed multiple myeloma (NDMM), but they were non-curative for most patients. The incorporation of daratumumab into these regimens, resulting in quadruplet therapies, has shown improved outcomes, though concerns over increased toxicity remain.

**Methods:**

In this systematic review and meta-analysis, we aimed to compare the efficacy and safety of daratumumab-incorporated quadruplet regimens versus traditional triplet regimens in NDMM. A search of PubMed, EMBASE, and the Cochrane Library identified six randomized controlled trials (RCTs) with 3,056 patients. Outcomes included response rates, minimal residual disease (MRD) negativity rate, progression-free survival (PFS), and adverse events.

**Results:**

Compared with triplet regimens, daratumumab-incorporated quadruplet combinations achieved a higher overall survival rate (ORR) (pooled OR = 2.36, 95% CI: 1.56-3.56, P < 0.0001), rate of complete response (CR) or better (pooled OR = 2.35, 95% CI: 1.99-2.77, P < 0.0001), very good partial response (VGPR) or better (pooled OR = 2.58, 95% CI: 1.76-3.79, P < 0.0001) and MRD negativity (pooled OR = 3.55, 95% CI: 2.54-4.96, P < 0.0001). The addition of daratumumab to triplet regimens significantly improved PFS compared with triplet regimens (pooled HR = 0.45, 95% CI: 0.39–0.52, P < 0.0001). Regarding safety, quadruplet regimens were associated with a higher incidence of lymphopenia, upper respiratory tract infection, pyrexia, and pneumonia.

**Conclusion:**

Incorporating daratumumab into backbone triplet regimens is associated with improved response rates, deeper remission and prolonged PFS with acceptable toxicity profile in patients with NDMM.

**Systematic review registration:**

https://inplasy.com/inplasy-2024-12-0026/, identifier INPLASY2024120026.

## Introduction

Triplet regimens, including bortezomib-lenalidomide-dexamethasone (VRd) and bortezomib-melphalan-prednisone (VMP), were widely approved induction treatments for multiple myeloma (MM) (1–3). For transplantation-eligible patients with newly diagnosed multiple myeloma (NDMM), the standard approach involved VRd induction therapy followed by autologous stem-cell transplantation, consolidation therapy with VRd, and maintenance therapy with lenalidomide (4). However, this strategy was noncurative in the vast majority of patients.

Quadruplet combinations, including daratumumab, have been investigated in the induction therapy of NDMM to achieve deeper remissions and prolonged survival (5, 6). Currently, the incorporation of daratumumab into standard triplet regimens has become the most widely used quadruplet strategy, replacing traditional triplet combinations as the standard of care (7–9). Daratumumab, a CD38-targeted human IgGκ monoclonal antibody, exerts its antitumor effects through multiple mechanisms (10). These include direct tumor cell killing via antibody-dependent cellular cytotoxicity (ADCC), complement-dependent cytotoxicity (CDC), and apoptosis induction (10). Additionally, daratumumab modulates the immune system by depleting immune-suppressive cells, such as regulatory T cells and myeloid-derived suppressor cells (MDSCs), and by enhancing the activity of effector immune cells like natural killer (NK) cells and T cells (10–12). Through these mechanisms, daratumumab not only directly targets malignant plasma cells but also reactivates the immune system to enhance antitumor responses (10–12). Commonly used quadruplet combinations include daratumumab-bortezomib-lenalidomide-dexamethasone (D-VRd), daratumumab-bortezomib-thalidomide-dexamethasone (D-VTd), and daratumumab-bortezomib-melphalan-prednisone (D-VMP). In the phase 2 GRIFFIN trial, the incorporation of daratumumab into the VRd regimen (D-VRd) led to improved outcomes in patients with NDMM. Stringent complete response (sCR) rates were higher for D-VRd compared with VRd (62.6% vs. 45.4%; P = 0.0177), minimal residual disease (MRD) negativity rates (10–^5^ threshold) were higher for D-VRd (51.0% vs. 20.4%; P < 0.0001), and the respective 24-month progression-free survival (PFS) rates were 95.8% (D-VRd) versus 89.8% (VRd) (13, 14). However, some studies have shown that quadruplet regimens do not offer superior survival outcomes compared to triplet regimens. The phase 2 AmaRC 03–16 trial did not show a significant benefit in PFS for the D-VCD (daratumumab-bortezomib-cyclophosphamide-dexamethasone) arm compared to the VCD (bortezomib-cyclophosphamide-dexamethasone) arm (15). In the phase 3 ALCYONE study, the D-VMP group did not demonstrate improved PFS compared to the VMP group in the high cytogenetic risk subgroup (HR [95% CI]: 0.78 [0.43, 1.43]) (16, 17). Additionally, the addition of daratumumab may increase the incidence of adverse events (18). The PERSEUS study demonstrated that, compared to the VRd group, the D-VRd group had higher rates of neutropenia (69.2% in the D-VRd group and 58.8% in the VRd group), thrombocytopenia (48.4% vs. 34.3%), and pneumonia (18.2% vs. 11.0%) (18).

Owing to uncertainties in therapeutic efficacy—as indicated by findings from the AmaRC 03-16 (15) and the high cytogenetic risk subgroup of the ALCYONE study (16, 17)—and the elevated incidence of hematologic toxicities (e.g., neutropenia, thrombocytopenia) and infections (e.g., pneumonia) (18), some experts remain cautious about universally recommending quadruplet regimens for all NDMM patients, resulting in a lack of definitive consensus in clinical practice. We propose that aggregating outcomes from different clinical trials may resolve this debate. This meta-analysis was designed to compare the efficacy and safety of daratumumab-incorporated quadruplet regimens and standard triplet therapies in patients with NDMM.

## Materials and methods

### Search strategy

Two independent authors conducted a comprehensive search for relevant information using the PubMed, EMBASE, and Cochrane Library databases. Only published trials with full-text papers were included. We also manually reviewed reference lists from eligible studies to identify additional relevant records. All available research published up to March 2025 was collected. The detailed search strategy is provided in supplementary information.

### Selection criteria

The studies identified were independently evaluated by two reviewers. Studies were included if they met the following inclusion criteria:

- research design: randomized controlled trials (RCTs);
- participants: patients with NDMM;
- intervention: daratumumab-incorporated quadruplet regimens versus triplet regimens;
- outcomes: overall response rate (ORR), the rate of complete response (CR) or better (comprising CR and sCR), the rate of very good partial response (VGPR) or better (comprising VGPR, CR and sCR), the rate of negative status for MRD (10–^5^ threshold), PFS and toxicity events.

### Data extraction

Data extraction from the included RCTs was performed by two researchers, covering detailed medication regimens, drug dosages, efficacy data, survival data, and incidence of toxicities.

### Methodological quality appraisal

Methodological quality of each study was assessed by two independent researchers. We adopted the Cochrane Collaboration Risk of Bias tool (19) to judge the quality of RCTs.

### Outcomes assessments

One objective was to compare the ORR, rate of CR or better, rate of VGPR or better, rate of negative MRD status and PFS between the two arms. Another objective was to assess the differences in safety outcomes between the two arms, including neutropenia, thrombocytopenia, anemia, lymphopenia, fatigue, upper respiratory tract infection, peripheral sensory neuropathy, diarrhea, constipation, nausea, pyrexia, peripheral edema, and pneumonia.

### Statistical analysis

Analyses were conducted using RevMan 5.4. Heterogeneity across the included trials was assessed using the I² statistic. An I² value of 25% to 50% was considered to indicate low heterogeneity, 50% to 75% moderate heterogeneity, and greater than 75% high heterogeneity. A random effects model was applied when the I² value exceeded 50%, whereas a fixed effects model was used otherwise.

## Results

### Selection of the trials

**Figure 1:**
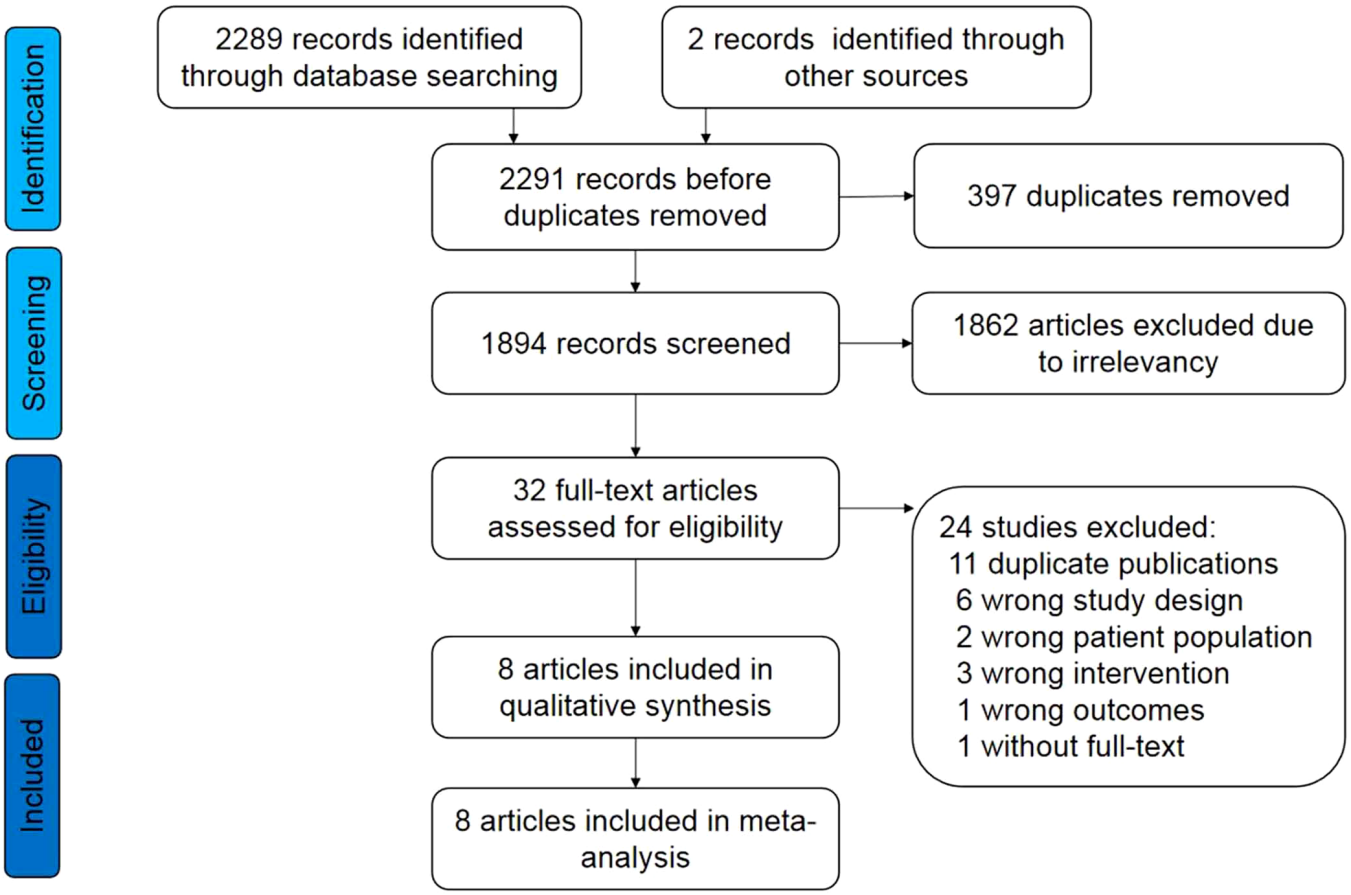
Flowchart of literature search and study selection.

The process of literature retrieval, selection, and identification is illustrated in **Figure 1**. Following the initial search, 2291 records were identified. Of these, 8 publications (13–18, 20, 21) of 6 RCTs met the inclusion criteria. Consequently, a total of 3,056 patients were included in this study.

### Characteristics of the trials

**Figure 2:**
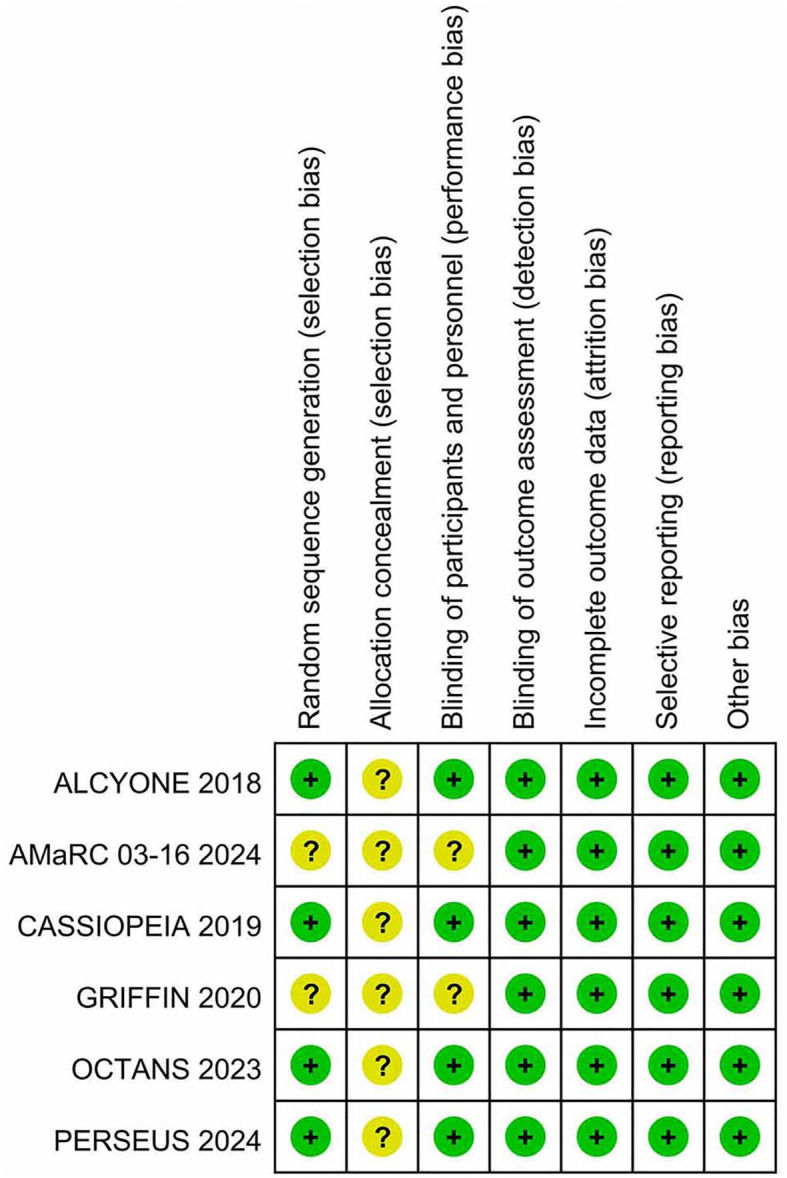
Risk of bias summary for RCTs.

**Figure 3:**
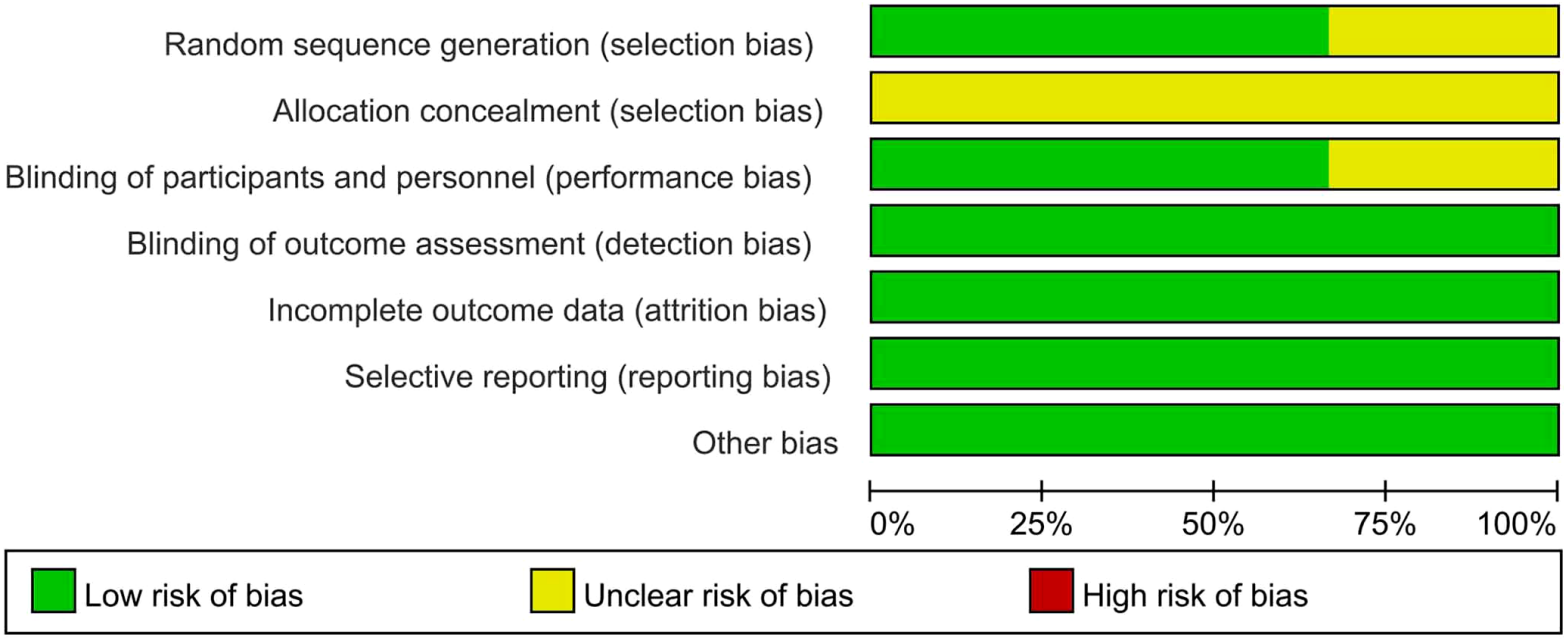
Risk of bias graph for RCTs.

**Table 1** presents the primary characteristics of the 6 RCTs. Each study had a complete full-text article available, and all RCTs included in the analysis were assessed as high quality. The quality appraisal of the 6 RCTs are shown in **Figures 2** and **3**.

**Table 1:** Attributes of studies meeting the inclusion criteria for the meta-analysis.

Clinical trials	Year	Number of patients	Median age (y)	Aera	Patients	Study arms		Study design
						Daratumumab-based quadruple-drug regimens	Standard triple-drug regimens	
ALCYONE	2018	706	71	Global	transplant-ineligible NDMM	D-VMP (daratumumab, bortezomib, melphalan and prednisone)	VMP (bortezomib, melphalan and prednisone/dexamethasone)	phase 3 RCT
AMaRC 03-16	2024	129	75	Australia	transplant-ineligible NDMM	D-VCD (daratumumab, bortezomib, cyclophosphamide and dexamethasone)	VCD (bortezomib, cyclophosphamide and dexamethasone)	phase 2 RCT
CASSIOPEIA	2019	1085	59	European	transplant-eligible NDMM	D-VTd (daratumumab, bortezomib, thalidomide and dexamethasone)	VTd (bortezomib, thalidomide and dexamethasone)	phase 3 RCT
GRIFFIN	2020	207	60	United States	transplant-eligible NDMM	D-RVd (daratumumab, lenalidomide, bortezomib and dexamethasone)	RVd (lenalidomide, bortezomib and dexamethasone)	phase 2 RCT
OCTANS	2023	220	69	China	transplant-ineligible NDMM	D-VPM (daratumumab, bortezomib, melphalan and prednisone)	VPM (bortezomib, melphalan and prednisone/dexamethasone)	phase 3 RCT
PERSEUS	2024	709	60	Europe and Australia	transplant-eligible NDMM	D-VRd (daratumumab, bortezomib, lenalidomide, and dexamethasone)	VRd (bortezomib, lenalidomide, and dexamethasone)	phase 3 RCT

NDMM, newly diagnosed multiple myeloma; RCT, randomized controlled trial.

### Responses and the rate of negative status for MRD

**Figure 4:**
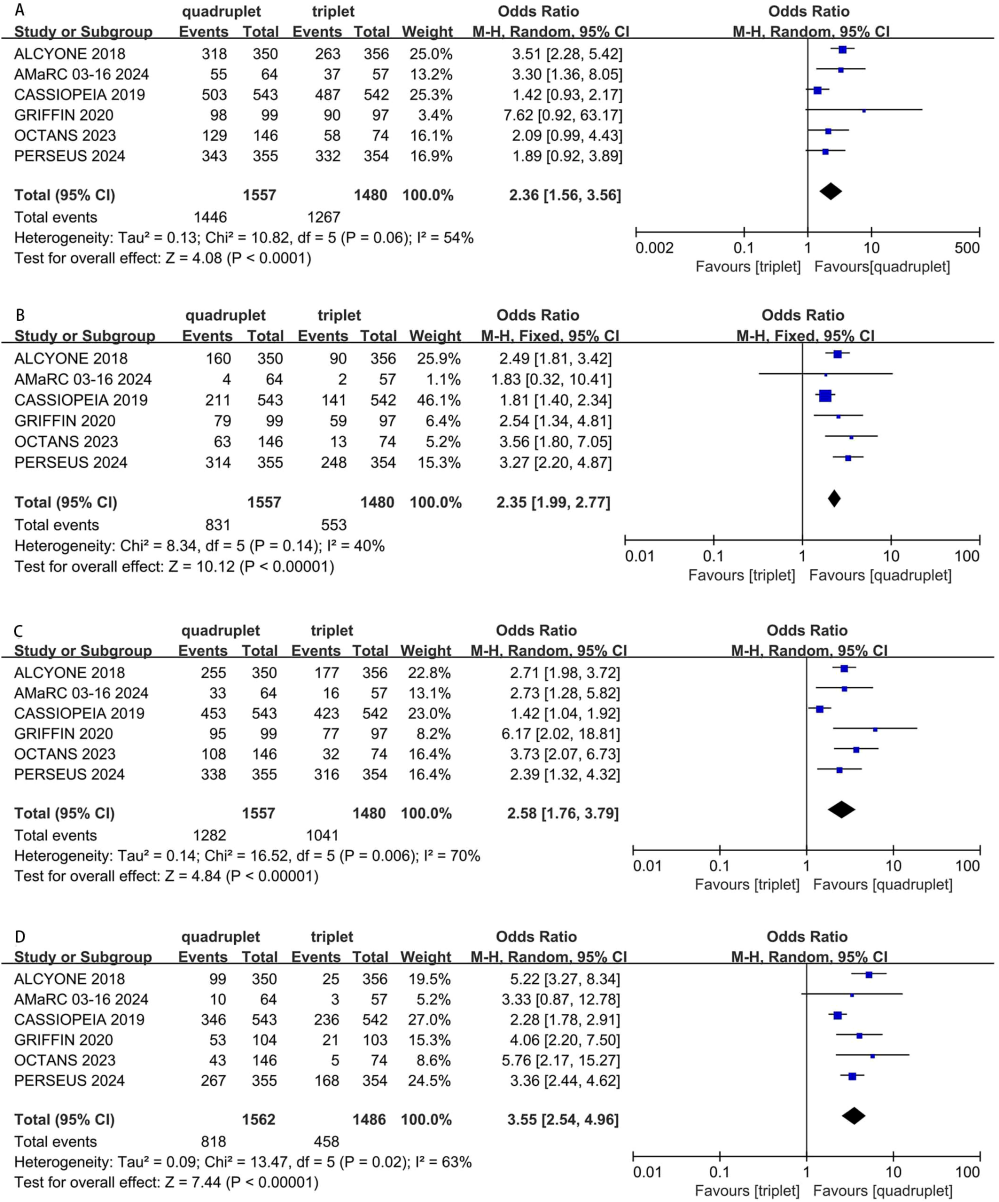
Forest plot of ORR **(A)**, CR or better **(B)**, VGPR or better **(C)** and MRD negativity **(D)** in daratumumab-incorporated quadruplet regimens versus standard triplet regimens.

Six studies were included in the analysis. Compared with triplet regimens, daratumumab-incorporated quadruplet regimens achieved a significantly higher ORR (pooled OR = 2.36, 95% CI: 1.56-3.56, P < 0.0001; moderate heterogeneity, P = 0.06, I² = 54%; **Figure 4A**), rate of CR or better (pooled OR = 2.35, 95% CI: 1.99-2.77, P < 0.0001; low heterogeneity, P = 0.14, I² = 40%; **Figure 4B**), rate of VGPR or better (pooled OR = 2.58, 95% CI: 1.76-3.79, P < 0.0001; moderate heterogeneity, P = 0.006, I² = 70%; **Figure 4C**), and rate of negative status for MRD (pooled OR = 3.55, 95% CI: 2.54-4.96, P < 0.0001; moderate heterogeneity, P = 0.02, I² = 63%; **Figure 4D**).

### PFS

**Figure 5:**
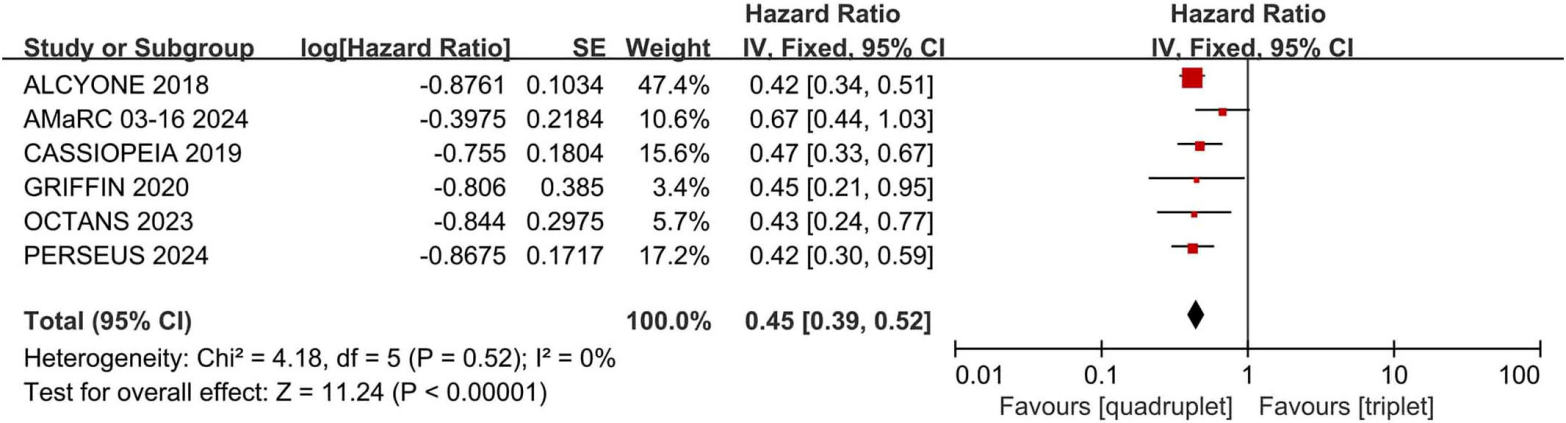
Forest plot of PFS in daratumumab-incorporated quadruplet regimens versus standard triplet regimens.

Compared with triplet regimens, daratumumab-incorporated quadruplet regimens achieved significantly improved PFS (pooled HR = 0.45, 95% CI: 0.39-0.52, P < 0.0001), with no heterogeneity (P = 0.52, I² = 0%; **Figure 5**).

### Adverse events

The results indicated that, compared with triplet regimens, daratumumab-incorporated quadruplet regimens had a higher incidence of adverse events, including any grade of lymphopenia (pooled OR = 1.33, 95% CI: 1.03-1.73, P = 0.03; low heterogeneity, P = 0.21, I² = 37%), lymphopenia grade ≥ 3 (pooled OR = 1.65, 95% CI: 1.24-2.20, P = 0.0006; low heterogeneity, P = 0.35, I² = 6%), any grade of upper respiratory tract infection (pooled OR = 1.78, 95% CI: 1.43-2.22, P < 0.0001; low heterogeneity, P = 0.26, I² = 25%), any grade of pyrexia (pooled OR = 1.23, 95% CI: 1.04-1.46, P = 0.01; low heterogeneity, P = 0.23, I² = 29%), any grade of pneumonia (pooled OR = 2.38, 95% CI: 1.76-3.24, P < 0.0001; moderate heterogeneity, P = 0.17, I² = 43%) and pneumonia grade ≥ 3 (pooled OR = 2.33, 95% CI: 1.62-3.35, P < 0.0001; no heterogeneity, P = 0.48, I² = 0%). The detailed data are shown in **Table 2**.

**Table 2:** Meta-analyses of adverse events.

Adverse events	Grade	Number of studies	Number of patients	Mode	Pooled RR (95%CI)	P value	I^2^	P for heterogeneity
Neutropenia	any grade	5	2888	R	1.40 (0.91-2.15)	0.12	84%	<0.0001
	grade ≥3	5	2888	R	1.44 (0.95-2.18)	0.08	83%	0.0001
Thrombocytopenia	any grade	5	2888	R	1.42 (0.99-2.02)	0.06	76%	0.003
	grade ≥3	5	2888	R	1.36 (0.94-1.98)	0.10	70%	0.009
Anemia	any grade	4	1814	F	0.85 (0.69-1.04)	0.11	47%	0.13
	grade ≥3	4	1814	F	0.87 (0.65-1.15)	0.32	0%	0.64
Lymphopenia	any grade	3	1490	F	1.33 (1.03-1.73)	0.03	37%	0.21
	grade ≥3	3	1490	F	1.65 (1.24-2.20)	0.0006	6%	0.35
Fatigue	any grade	3	1973	F	1.08 (0.89-1.31)	0.44	23%	0.27
	grade ≥3	3	1973	F	0.76 (0.44-1.32)	0.33	0%	0.44
Upper respiratory tract infection	any grade	4	1814	F	1.78 (1.43-2.22)	<0.0001	25%	0.26
	grade ≥3	4	1814	F	1.22 (0.60-2.47)	0.58	44%	0.15
Peripheral sensory neuropathy	any grade	4	2687	F	0.86 (0.73-1.00)	0.06	26%	0.25
	grade ≥3	4	2687	F	0.90 (0.64-1.25)	0.52	19%	0.29
Diarrhea	any grade	4	1814	F	1.18 (0.96-1.44)	0.11	0%	0.43
	grade ≥3	4	1814	F	1.24 (0.84-1.84)	0.28	0%	0.68
Constipation	any grade	4	2188	F	1.12 (0.94-1.33)	0.19	0%	0.40
	grade ≥3	4	2188	F	1.22 (0.60-2.48)	0.59	0%	0.84
Nausea	any grade	4	2673	F	1.19 (1.00-1.43)	0.05	0%	0.45
	grade ≥3	4	2673	F	1.50 (0.83-2.70)	0.18	0%	0.74
Pyrexia	any grade	5	2888	F	1.23 (1.04-1.46)	0.01	29%	0.23
	grade ≥3	5	2888	F	1.02 (0.59-1.74)	0.96	0%	0.98
Peripheral edema	any grade	3	1973	F	1.06 (0.87-1.30)	0.55	0%	0.72
	grade ≥3	3	1973	F	0.82 (0.34-1.99)	0.66	32%	0.23
Pneumonia	any grade	3	1613	F	2.38 (1.76-3.24)	<0.0001	43%	0.17
	grade ≥3	3	1613	F	2.33 (1.62-3.35)	<0.0001	0%	0.48

R: random effects model;F: fixed effects model.

### Subgroup analysis

We introduced the subgroup analyses for PFS regarding sex, age, race, ISS disease stage, type of multiple myeloma, cytogenetic risk, ECOG performance status, baseline creatinine clearance and baseline hepatic function. The detailed data are shown in **Table 3** and **Supplementary Figures 1**–**9**. Differing results were observed only in the MM patients with abnormal creatinine clearance. The quadruplet regimens did not achieve a better PFS (pooled HR = 0.59, 95% CI: 0.31-1.11; moderate heterogeneity, P = 0.06, I² = 64%) than triplet regimens in patients with baseline creatinine clearance ≤60ml/min.

**Table 3:** Subgroup analyses for PFS.

Subgroup		Number of Studies	Pooled HR (95% CI)	I^2^	Model
Sex	Male	5	0.56 (0.45, 0.68)	0%	F
	Female	5	0.40 (0.31, 0.52)	0%	F
Age	<75 yr	3	0.47 (0.37, 0.61)	0%	F
	≥75 yr	2	0.64 (0.44, 0.92)	30%	F
Race	White	2	0.50 (0.40, 0.63)	0%	F
	Other	2	0.29 (0.15, 0.57)	0%	F
ISS disease stage	I	4	0.53 (0.37, 0.75)	0%	F
	II	4	0.40 (0.31, 0.52)	0%	F
	III	4	0.52 (0.38, 0.70)	0%	F
Type of multiple myeloma	IgG	4	0.43 (0.35, 0.54)	0%	F
	Non-IgG	4	0.62 (0.43, 0.89)	0%	F
Cytogenetic risk	Standard	5	0.42 (0.34, 0.51)	0%	F
	High	5	0.65 (0.47, 0.88)	0%	F
ECOG performance status	0	5	0.44 (0.34, 0.57)	0%	F
	≥1	4	0.49 (0.40, 0.61)	0%	F
Baseline creatinine clearance	≤60ml/min	3	0.59 (0.31, 1.11)	64%	R
	>60ml/min	3	0.56 (0.43, 0.74)	39%	F
Baseline hepatic function	Normal	3	0.50 (0.41, 0.63)	0%	F
	Impaired	3	0.39 (0.23, 0.66)	0%	F

R, random effects model; F, fixed effects model.

### Heterogeneity analysis

Heterogeneity analysis was introduced in the primary outcomes: PFS, any grade of pneumonia and pneumonia grade ≥ 3. No statistically significant heterogeneity was detected. The results are shown in **Figure 5** and **Table 2**.

### Sensitivity analysis

We introduced the sensitivity analysis to assess the influence of each study on the pooled outcomes by removing single trial each time in primary outcomes, including PFS, any grade of pneumonia and pneumonia grade ≥ 3. No individual study substantially affected the pooled results. The results are shown in **Supplementary Table 1**-**Supplementary Table 3**.

## Discussion

Triplet regimens were the standard-of-care treatments in MM (22). Despite significant improvements in survival for MM patients with VRd and other triplet regimens, MM remains incurable (23). To achieve deeper remission or potential cure for MM, additional agents have been integrated into standard triplet regimens, forming quadruplet combinations. This meta-analysis was conducted to assess whether daratumumab-incorporated quadruplet regimens provide advantages over the standard triplet regimens in patients with NDMM. This study demonstrated that the incorporation of daratumumab into triplet regimens was associated with significantly improved ORR (pooled OR = 2.36, P < 0.0001), MRD negativity (pooled OR = 3.55, P < 0.0001), and PFS (pooled HR = 0.45, P < 0.0001). These findings are clinically significant and may assist clinicians in selecting the most effective anti-myeloma regimen for patients with NDMM.

In patients with NDMM across varying cytogenetic risks, the efficacy of daratumumab-incorporated quadruplet regimens in improving PFS compared with standard triplet regimens has been a subject of considerable research (16, 21). This question is particularly contentious for high cytogenetic risk patients, where the superiority of daratumumab incorporating remains uncertain (16, 21). Among the clinical studies we reviewed, only the PERSEUS (18) study demonstrated a significant improvement in PFS (HR [95% CI]: 0.59 [0.36, 0.99]) with D-VRd compared with VRd in high cytogenetic risk patients. In contrast, other studies, including ALCYONE (HR [95% CI]: 0.78 [0.43, 1.43]) (16, 17), CASSIOPEIA (HR [95% CI]: 0.67 [0.35, 1.30]) (21), AMaRC 03-16 (HR [95% CI]: 0.70 [0.25, 1.98]) (15) and OCTANS (HR [95% CI]: 0.34 [0.09, 1.32]) (20), did not report a PFS advantage with the quadruplet regimens. After conducting a meta-analysis of the available data, we found that the daratumumab-incorporated quadruplet regimens significantly improved PFS in patients with high cytogenetic risk. This analysis helps to clarify the conflicting results observed in previous trials, where some studies did not report a PFS benefit with the quadruplet regimens in high cytogenetic risk patients group. Our findings provide valuable clinical insights into the treatment strategy for high cytogenetic risk patients with NDMM.

The potential benefit of incorporating daratumumab to improve survival in elderly patients remains a subject of ongoing debate (24). Its inclusion may result in stronger immunosuppression, particularly pronounced in elderly patients, thereby increasing the risk of infections, fever, and other adverse events (24, 25). The higher incidence of adverse events could diminish the therapeutic advantage of daratumumab, potentially limiting its benefit on survival. In NDMM patients over 75 years old, the ALCYONE (16, 17) trial demonstrated that D-VMP resulted in better PFS than VMP, but the AMaRC 03-16 (15) trial did not show superior PFS with the D-VCD compared with VCD. Therefore, we conducted a subgroup analysis based on age. The results after pooling the data suggest that in elderly patients over 75 years old, daratumumab-incorporated quadruplet regimens resulted in longer PFS compared with standard triplet regimens. This finding provides important guidance for the treatment of elderly NDMM patients.

Based on its metabolism being independent of renal function and its role in rapidly reducing free light chains (FLCs), daratumumab is considered to have significant therapeutic advantages in MM patients with renal insufficiency (RI) (26–28). Previous meta-analysis demonstrated that addition of daratumumab to backbone regimens significantly improved PFS and OS in NDMM with RI (29). However, this study found that, in MM patients with baseline creatinine clearance ≤60ml/min, the addition of daratumumab did not achieve a better PFS. This may be attributed to the insufficient number of studies (only three clinical trials) included in the subgroup analysis of patients with renal dysfunction in our meta-analysis.

The adverse events of the quadruplet regimens are a major concern for clinicians. Our meta-analysis confirmed that the addition of daratumumab results in an increased rate of lymphopenia. Since CD38 is not only expressed on the surface of myeloma cells but also on some B lymphocytes, daratumumab may lead to lymphopenia (10). The reduction of lymphocytes will further result in immunosuppression, thus increasing the chance of infection. This may explain why quadruplet regimens had higher incidence of upper respiratory tract infection, pneumonia and pyrexia than triplet. These findings suggest that clinicians should pay more attention to the immune system and infections of patients when the quadruplet regimens were adopted.

This study has several limitations. First, due to the inability to obtain relevant data, we are unable to assess whether the addition of daratumumab would result in an OS benefit. Second, although we aimed to evaluate the association between daratumumab and survival in patients with specific high-risk cytogenetic abnormalities, such as del(17p) or P53 mutations, this analysis could not be completed due to the lack of relevant data. Third, due to the limited number of included studies, Begg’s and Egger’s tests could not be used to reliably assess publication bias.

## Conclusion

The present study suggests that incorporating daratumumab into backbone triplet regimens is associated with improved response rates, deeper remission and prolonged PFS with acceptable safety in patients with NDMM.

## Data Availability

The raw data supporting the conclusions of this article will be made available by the authors, without undue reservation.

## Supplementary material

Supplementary Figure 1 Subgroup analyses for PFS regarding sex.

Supplementary Figure 2 Subgroup analyses for PFS regarding age.

Supplementary Figure 3 Subgroup analyses for PFS regarding race.

Supplementary Figure 4 Subgroup analyses for PFS regarding ISS disease stage.

Supplementary Figure 5 Subgroup analyses for PFS regarding type of multiple myeloma.

Supplementary Figure 6 Subgroup analyses for PFS regarding cytogenetic risk.

Supplementary Figure 7 Subgroup analyses for PFS regarding ECOG performance status.

Supplementary Figure 8 Subgroup analyses for PFS regarding baseline creatinine clearance.

Supplementary Figure 9 Subgroup analyses for PFS regarding baseline hepatic function.
