# Supplementary material for: Addition of daratumumab to standard triplet regimens achieved better survival in newly diagnosed multiple myeloma: a systematic review and meta-analysis of randomized controlled trials
Source: Front Oncol. 2025 Oct 30;15:1619115. doi: 10.3389/fonc.2025.1619115 (PMC12611664; doi:10.3389/fonc.2025.1619115)
Supplement: Supplementary file 1 [file Table1.docx]

Supplementary Table 1. Sensitivity analyses for PFS by excluding each study.

| Excluded studies | P value | HR (95 %CI) |
| --- | --- | --- |
| ALCYONE 2018 | ＜0.00001 | 0.48 (0.40, 0.58) |
| AMaRC 03-16 2024 | ＜0.00001 | 0.43 (0.37, 0.50) |
| CASSIOPEIA 2019 | ＜0.00001 | 0.45 (0.38, 0.52) |
| GRIFFIN 2020 | ＜0.00001 | 0.45 (0.39, 0.52) |
| OCTANS 2023 | ＜0.00001 | 0.45 (0.39, 0.52) |
| PERSUS 2024 | ＜0.00001 | 0.46 (0.39, 0.53) |

Supplementary Table 2. Sensitivity analyses for any grade of pneumonia by excluding each study.

| Excluded studies | P value | OR (95 %CI) |
| --- | --- | --- |
| ALCYONE 2018 | 0.0002 | 1.98 (1.37, 2.86) |
| OCTANS 2023 | ＜0.00001 | 2.37 (1.68, 3.33) |
| PERSEUS 2024 | ＜0.00001 | 3.08 (1.99, 4.78) |

Supplementary Table 3. Sensitivity analyses for pneumonia grade ≥ 3 by excluding each study.

| Excluded studies | P value | OR (95 %CI) |
| --- | --- | --- |
| ALCYONE 2018 | 0.002 | 2.00 (1.28, 3.14) |
| OCTANS 2023 | ＜0.0001 | 2.32 (1.53, 3.52) |
| PERSEUS 2024 | ＜0.0001 | 2.76 (1.70, 4.48) |
