# Supplementary material for: Addition of daratumumab to standard triplet regimens achieved better survival in newly diagnosed multiple myeloma: a systematic review and meta-analysis of randomized controlled trials
Source: Front Oncol. 2025 Oct 30;15:1619115. doi: 10.3389/fonc.2025.1619115 (PMC12611664; doi:10.3389/fonc.2025.1619115)
Supplement: Supplementary file 2 [file DataSheet1.docx]

**Search strategy and terms in PubMed database**

Research question as searched in PubMed on 05-03-2025

**Daratumumab**

(((((((((daratumumab[Title/Abstract]) OR Darzalex[Title/Abstract]) OR human CD38[Title/Abstract]) OR human-CD38[Title/Abstract]) OR humax CD38[Title/Abstract]) OR humax-CD38[Title/Abstract]) OR antiCD38[Title/Abstract]) OR anti CD38[Title/Abstract]) OR anti-CD38[Title/Abstract])

**Multiple myeloma**

("Multiple Myeloma"[Mesh]) OR (((((((((((((((((((Multiple Myelomas[Title/Abstract]) OR Myelomas, Multiple[Title/Abstract]) OR Myeloma, Multiple[Title/Abstract]) OR Myeloma, Plasma-Cell[Title/Abstract]) OR Myeloma, Plasma Cell[Title/Abstract]) OR Myelomas, Plasma-Cell[Title/Abstract]) OR Plasma-Cell Myeloma[Title/Abstract]) OR Plasma-Cell Myelomas[Title/Abstract]) OR Myelomatosis[Title/Abstract]) OR Myelomatoses[Title/Abstract]) OR Plasma Cell Myeloma[Title/Abstract]) OR Cell Myeloma, Plasma[Title/Abstract]) OR Cell Myelomas, Plasma[Title/Abstract]) OR Myelomas, Plasma Cell[Title/Abstract]) OR Plasma Cell Myelomas[Title/Abstract]) OR Kahler Disease[Title/Abstract]) OR Disease, Kahler[Title/Abstract]) OR Myeloma-Multiple[Title/Abstract]) OR Myeloma Multiple[Title/Abstract])

**Study**

("randomized controlled trial"[pt] OR "controlled clinical trial"[pt] OR randomized[tiab] OR placebo[tiab] OR "drug therapy"[sh] OR randomly[tiab] OR trial[tiab] OR groups[tiab]) NOT (Meta-Analysis[ptyp] OR Review[ptyp] OR systematic[sb])

**Limitations**

((rat OR Rats OR Mouse OR Mice OR pig OR pigs OR cow OR cows OR sheep OR chicken* OR dog OR dogs) NOT human [mesh])

**Combine:** (“Daratumumab” AND “Multiple myeloma” AND “Study”) NOT Limitations
