# Supplementary figures and images for: Addition of daratumumab to standard triplet regimens achieved better survival in newly diagnosed multiple myeloma: a systematic review and meta-analysis of randomized controlled trials
Source: Front Oncol. 2025 Oct 30;15:1619115. doi: 10.3389/fonc.2025.1619115 (PMC12611664; doi:10.3389/fonc.2025.1619115)

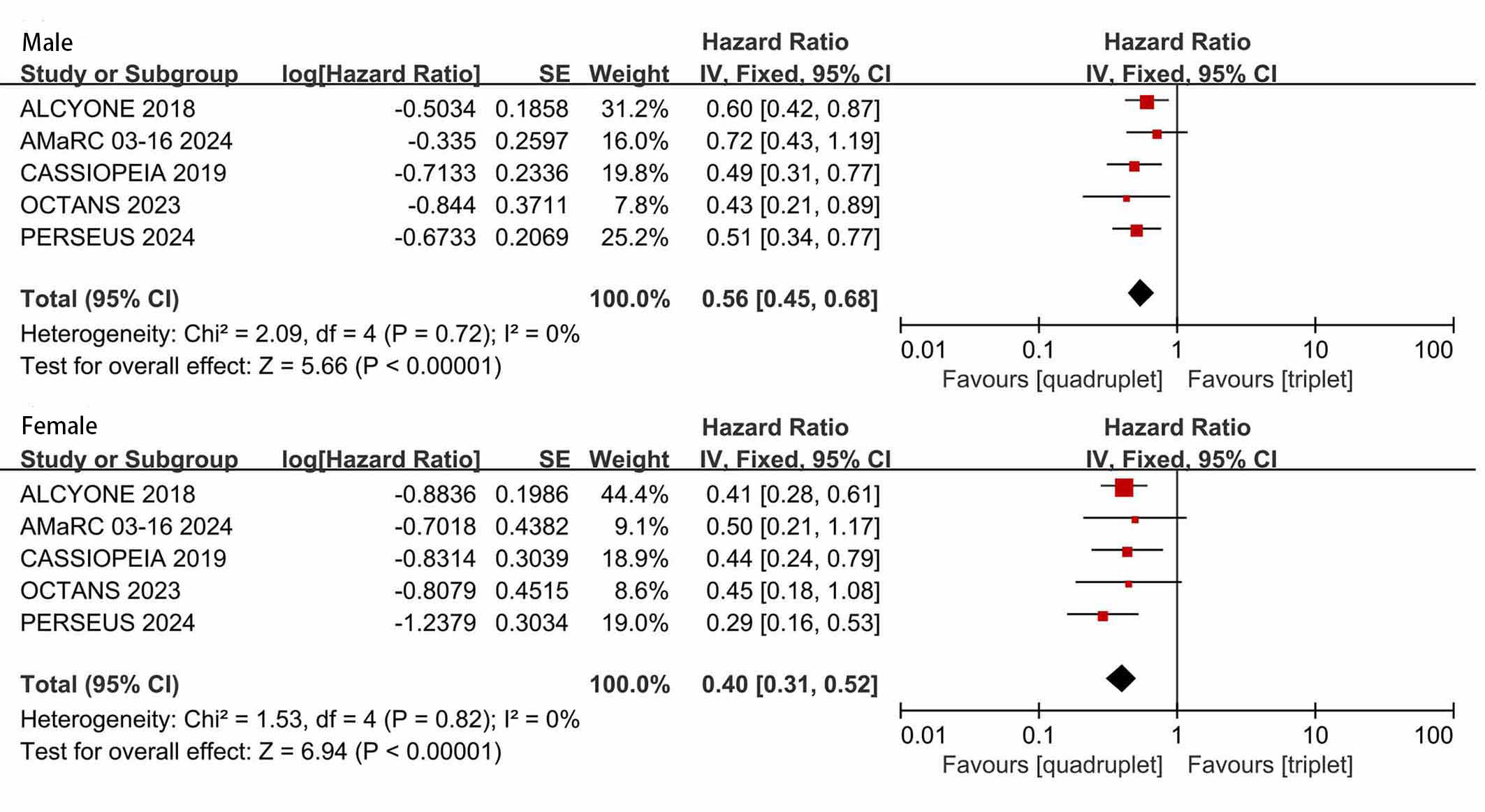

Supplement: Supplementary Figure 1 — Subgroup analyses for PFS regarding sex. [file Image1.tif]

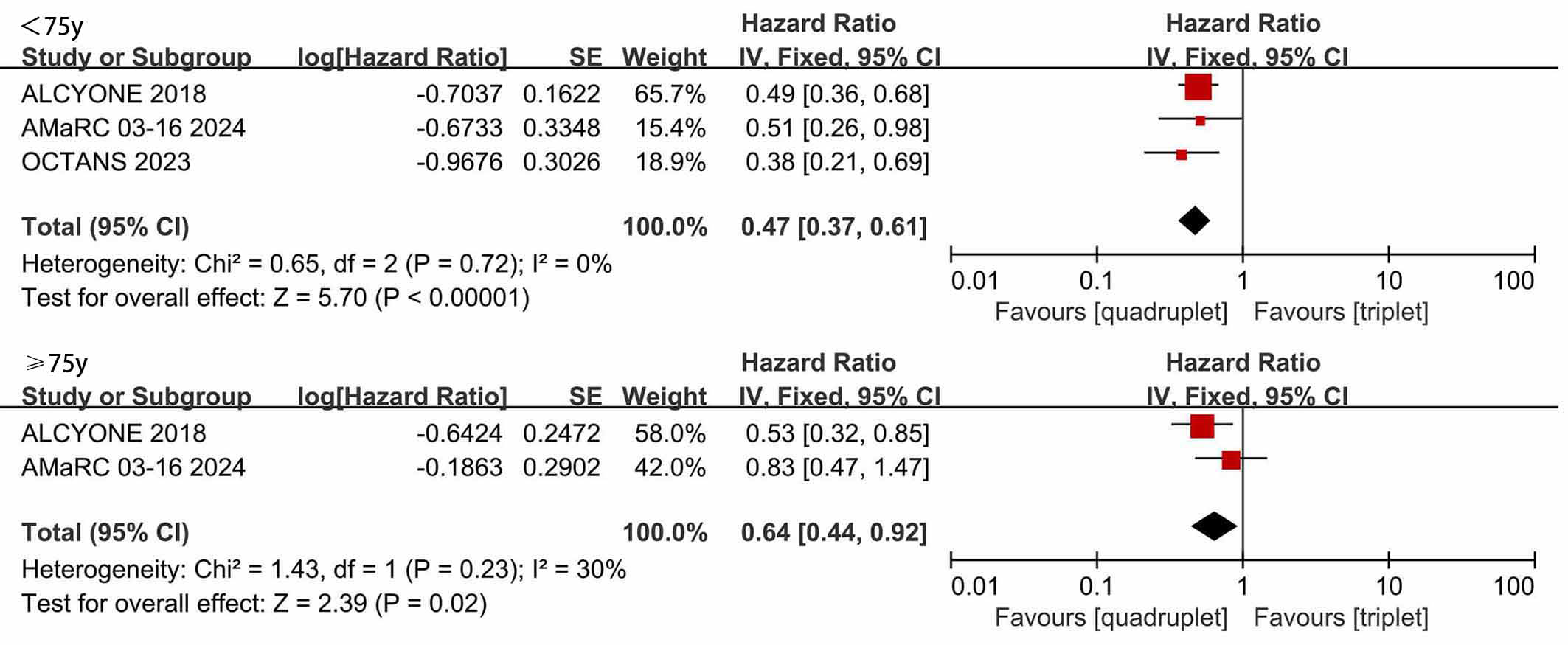

Supplement: Supplementary Figure 2 — Subgroup analyses for PFS regarding age. [file Image2.tif]

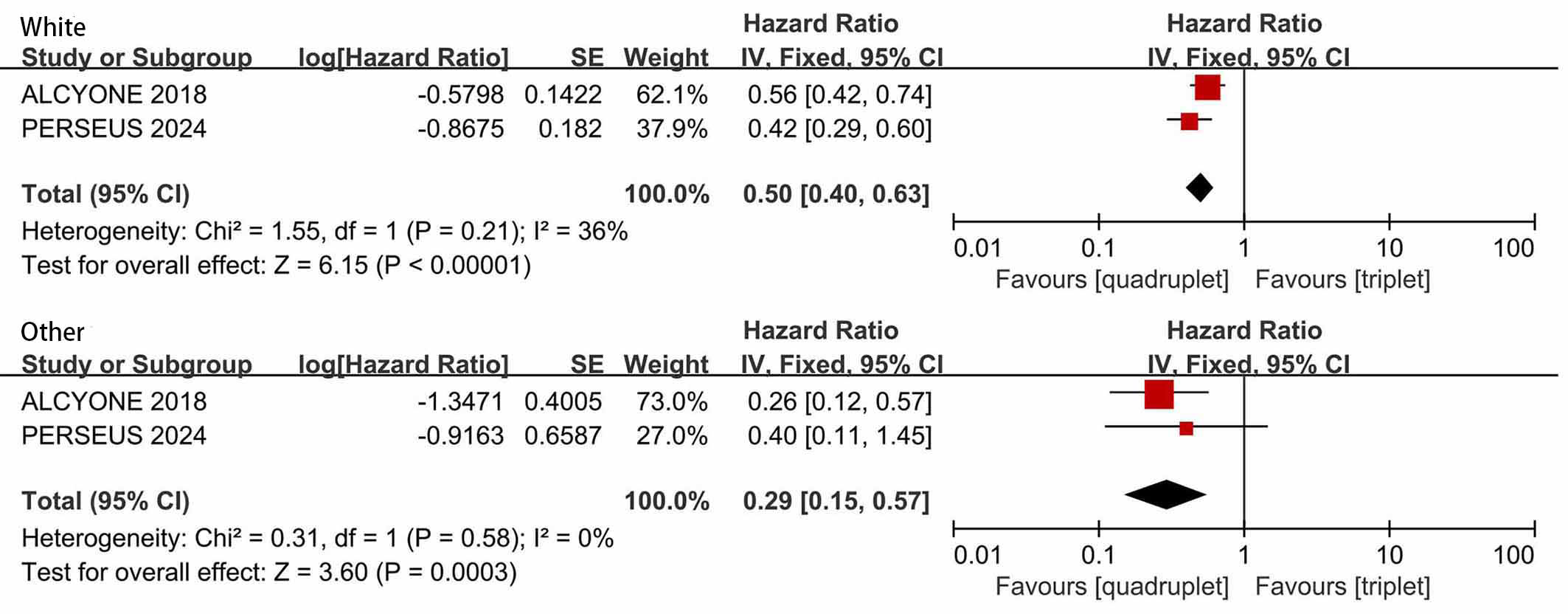

Supplement: Supplementary Figure 3 — Subgroup analyses for PFS regarding race. [file Image3.tif]

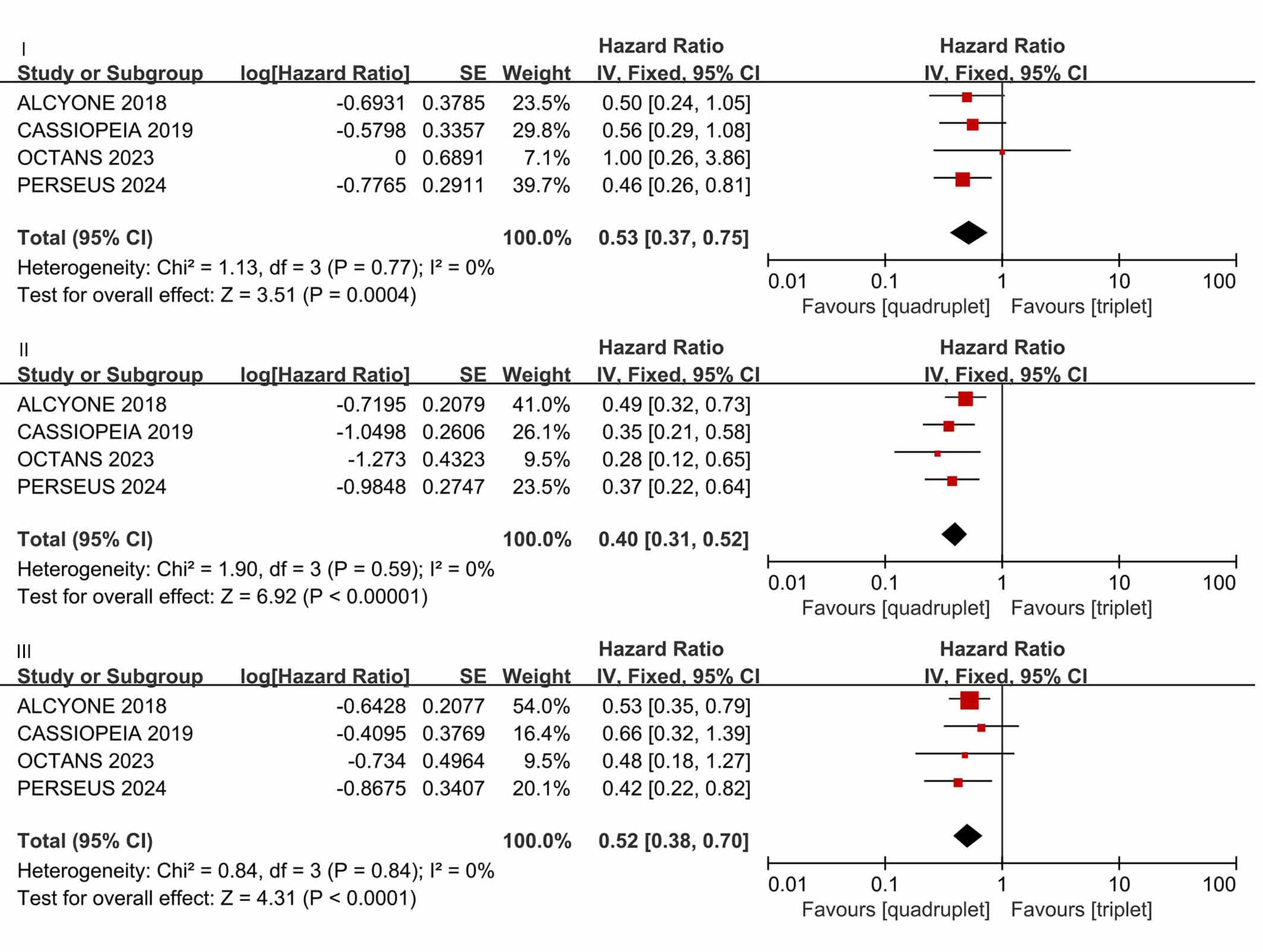

Supplement: Supplementary Figure 4 — Subgroup analyses for PFS regarding ISS disease stage. [file Image4.tif]

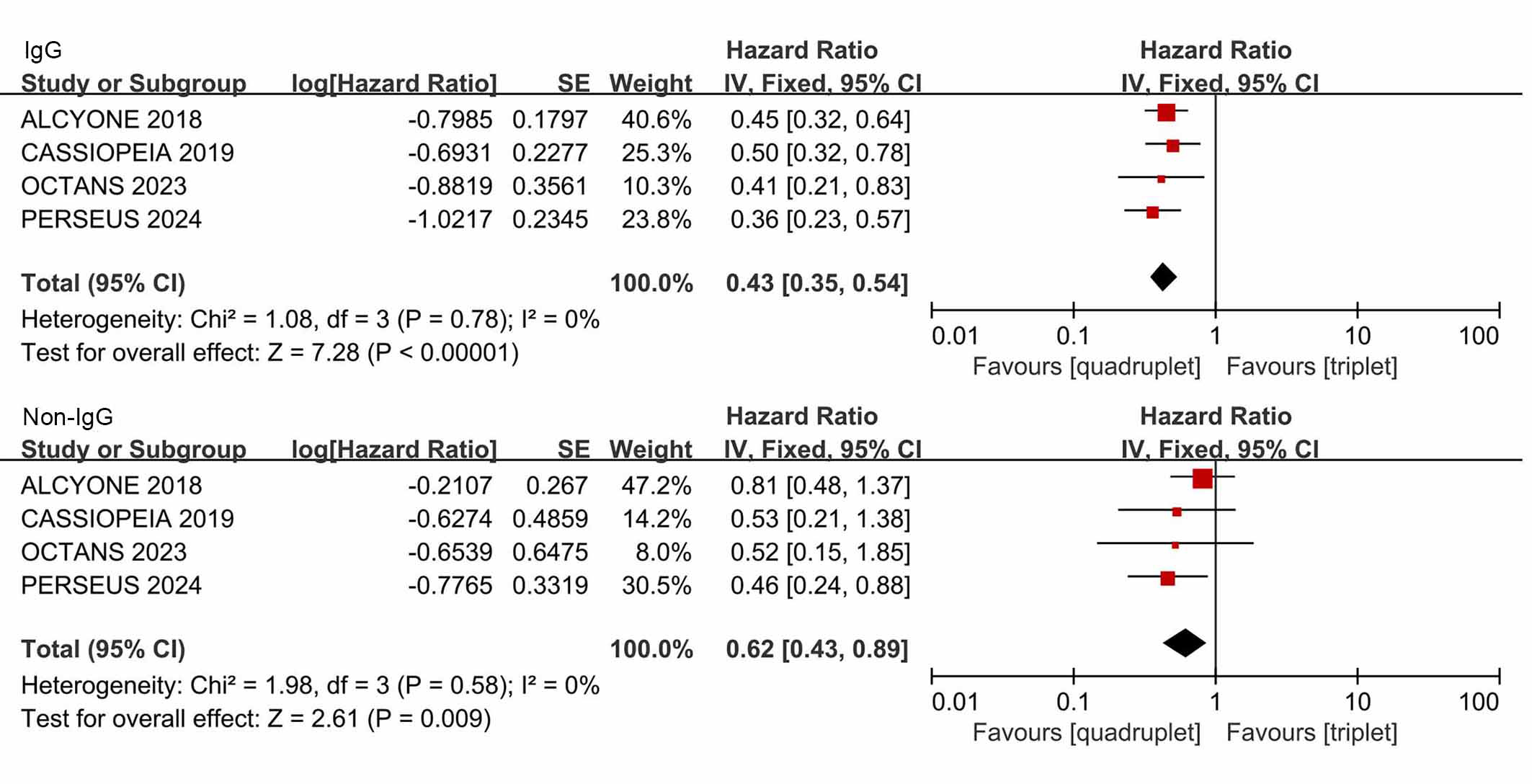

Supplement: Supplementary Figure 5 — Subgroup analyses for PFS regarding type of multiple myeloma. [file Image5.tif]

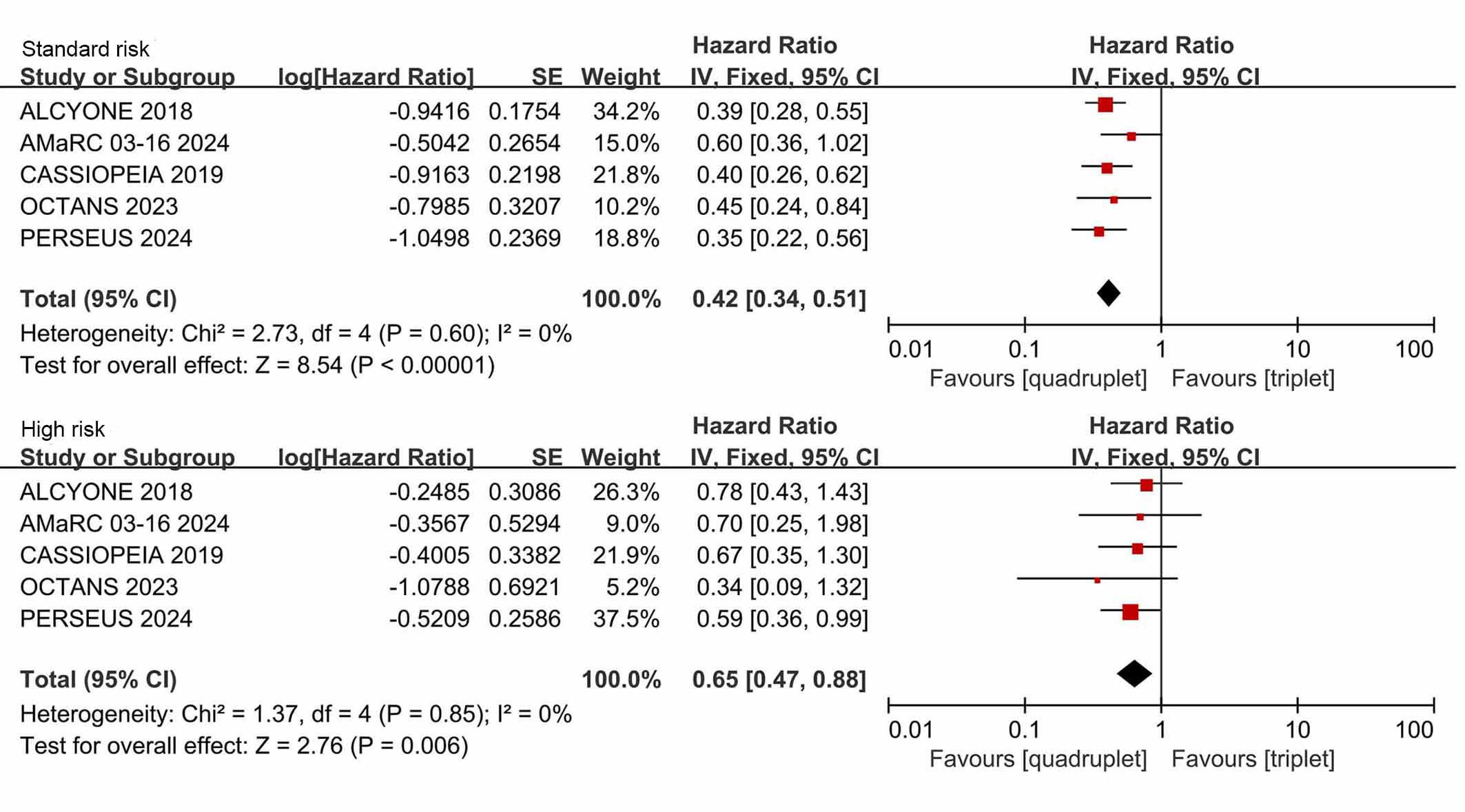

Supplement: Supplementary Figure 6 — Subgroup analyses for PFS regarding cytogenetic risk. [file Image6.tif]

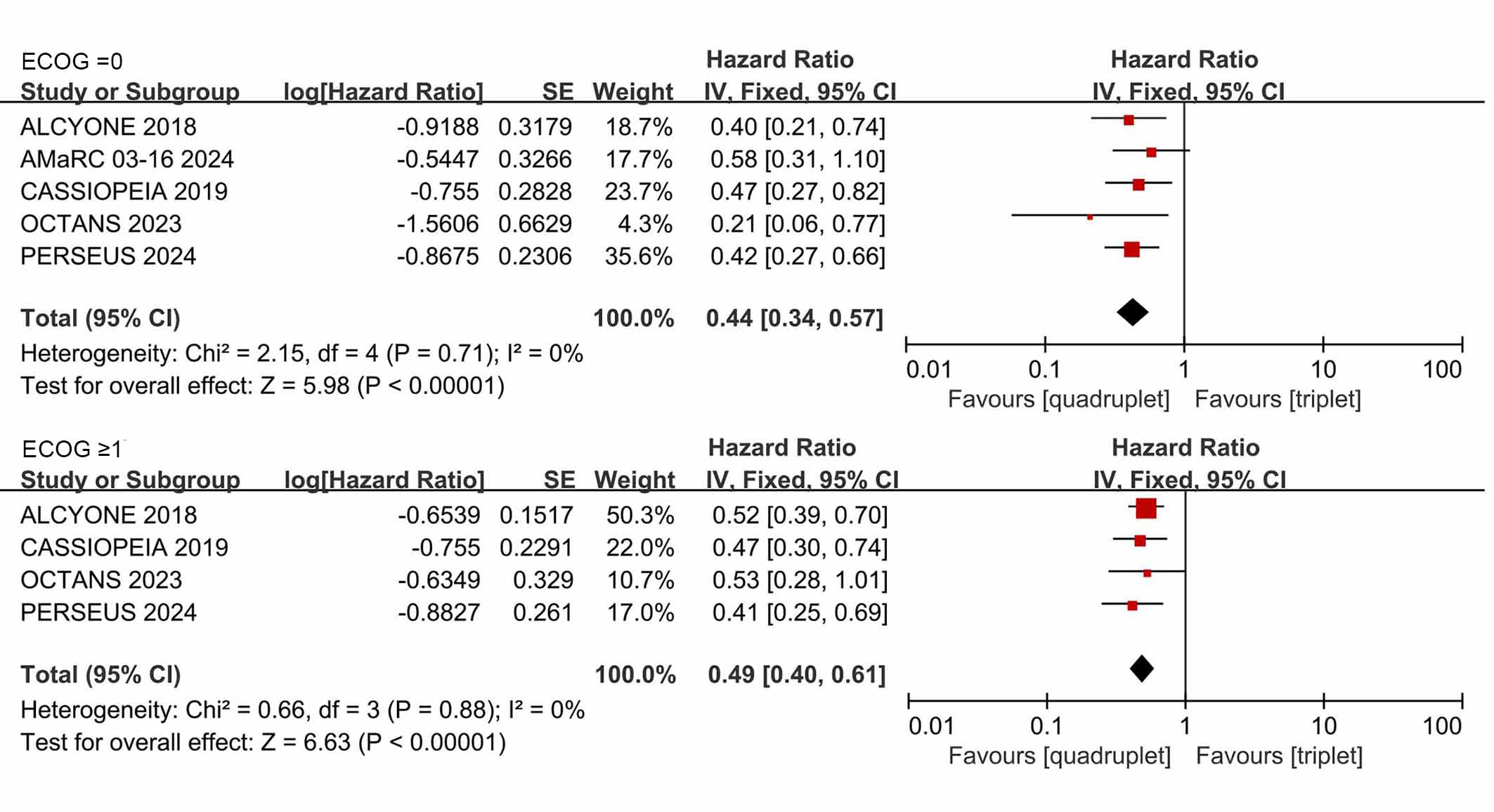

Supplement: Supplementary Figure 7 — Subgroup analyses for PFS regarding ECOG performance status. [file Image7.tif]

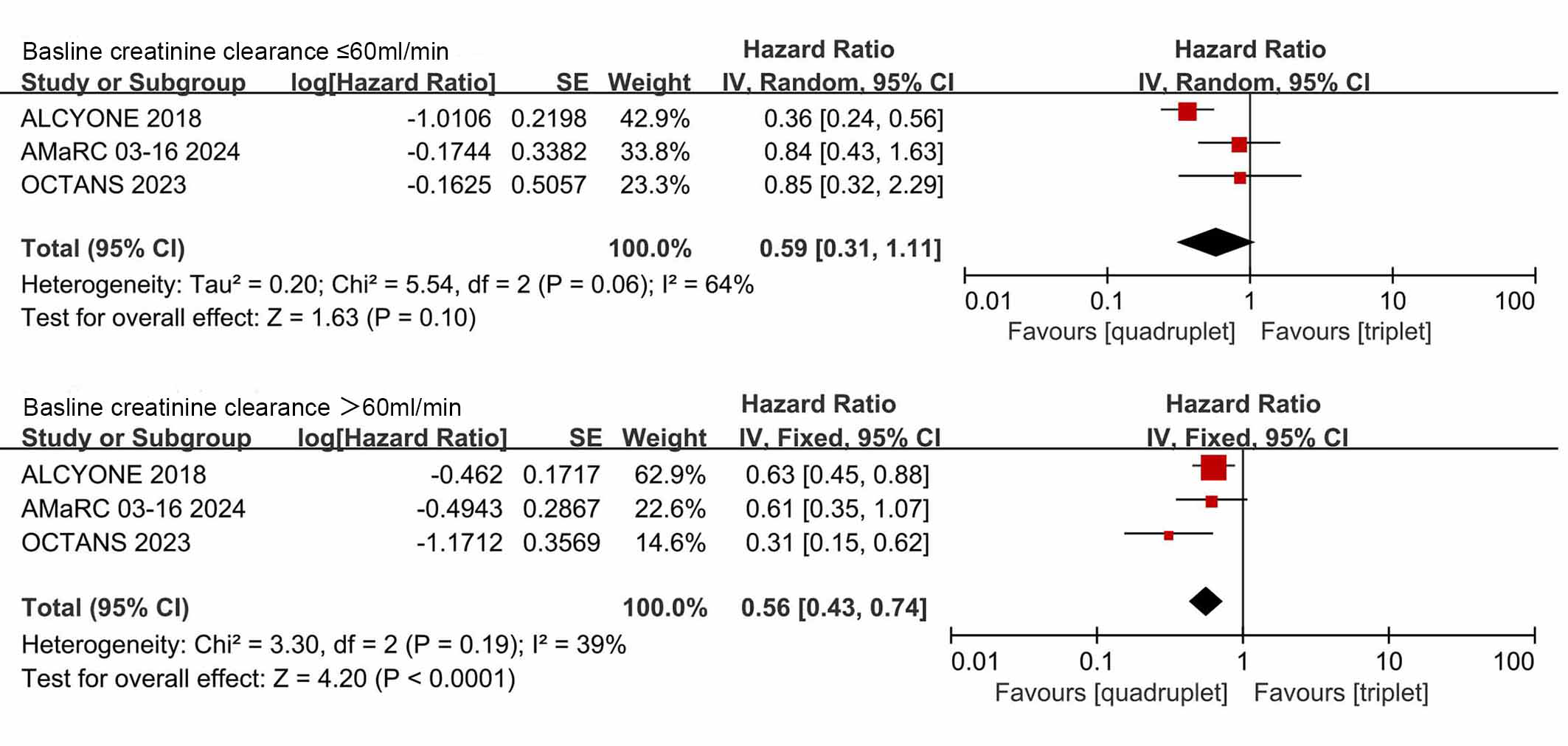

Supplement: Supplementary Figure 8 — Subgroup analyses for PFS regarding baseline creatinine clearance. [file Image8.tif]

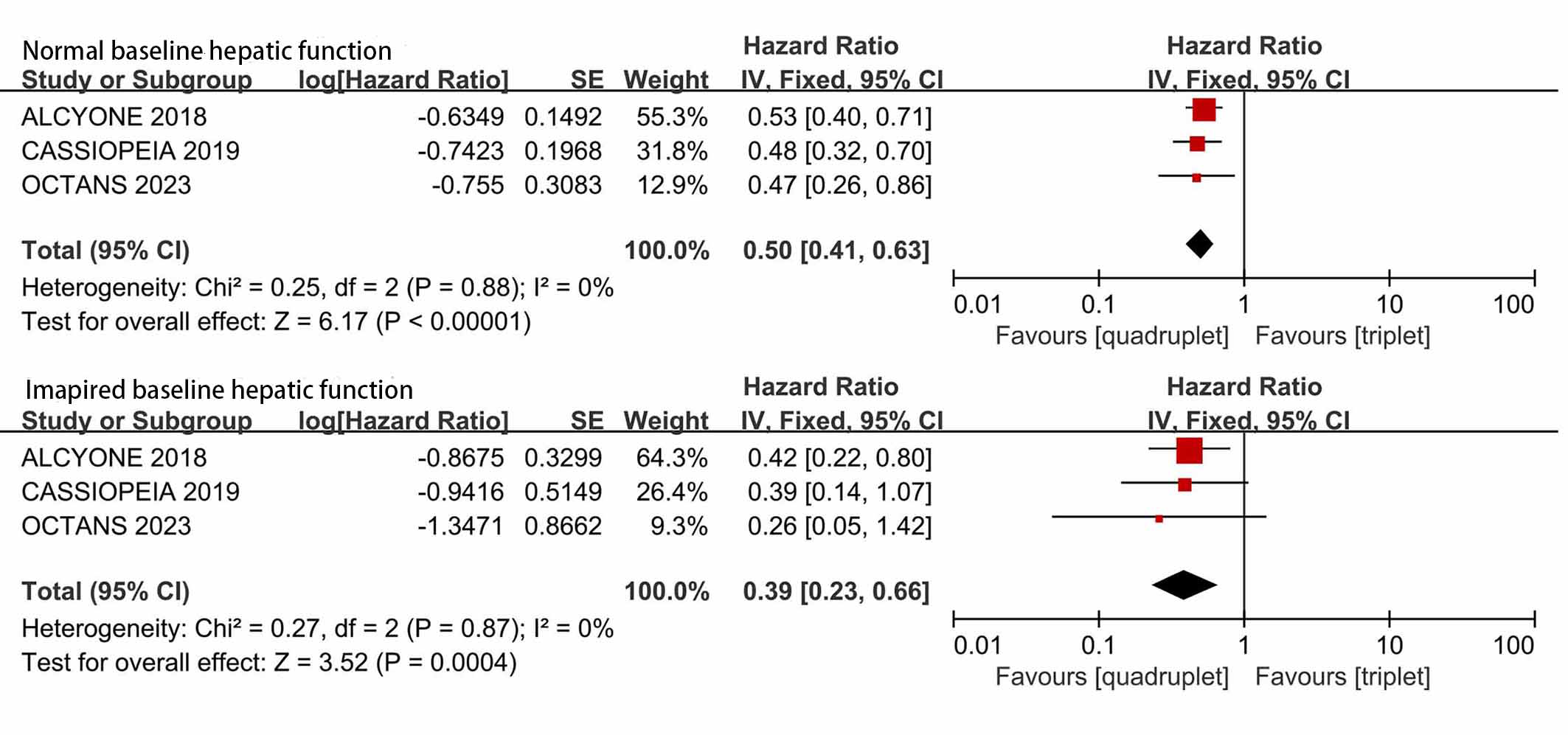

Supplement: Supplementary Figure 9 — Subgroup analyses for PFS regarding baseline hepatic function. [file Image9.tif]
